# 色谱在药物-机体复杂巨系统研究中的应用进展

**DOI:** 10.3724/SP.J.1123.2021.06021

**Published:** 2021-09-08

**Authors:** Pu JIA, Yangyang BIAN, Yajun BAI, Xue MENG, Shuomo GAO, Ye ZHAO, Yujie CAI, Xiaohui ZHENG

**Affiliations:** 1.西北大学, 陕西 西安 710069; 1. Northwest University, Xi’an 710069, China; 2.陕西省中医药研究院, 陕西 西安 710004; 2. Shaanxi Academy of Tradition Chinese Medicine, Xi’an 710004, China; 3.江南大学, 江苏 无锡 214122; 3. Jiangnan University, Wuxi 214122, China

**Keywords:** 色谱, 分离分析, 辨识技术, 药物-机体复杂巨系统, 创新药物, 综述, chromatography, separation and analysis, identification technology, giant complex drug-organism system, innovative drugs, review

## Abstract

色谱是一门以分离分析为主,旨在追求复杂事物纯而净的分析化学的重要分支学科。其经过百余年的发展,理论与技术日臻完善,集科学、技术与艺术于一体。近年来,色谱及其与质谱、核磁共振波谱、原子发射光谱等联用技术极大推动了环境、食品、石油化工、生物医药等领域中所涉及复杂体系的研究进展。作为我国传统文化的核心代表,中医药为中国乃至世界人民的健康服务逾千年,从古至今历经上千年临床考验,疗效经久不衰。近年来,中国政府强调继承与创新,加大推进中医药的现代化与国际化。然而中药自身的多成分协同起效复杂性及其与机体时刻新陈代谢变化的复杂性往往相互作用,由此形成了药物-机体复杂巨系统。该复杂巨系统的分析研究是中医药现代化进程的关键瓶颈。色谱的优势在于复杂成分的分离与分析,此恰能为上述复杂巨系统提供技术支撑,色谱及其联用技术已成为推动中医药分子化、数字化、信息化乃至现代化的主流技术。该文综述了色谱及其联用技术在中药复杂体系、复杂生命过程及药物-机体复杂巨系统中的应用进展,介绍了笔者研究团队对中医药现代化的认识、研究思路和研究工作,最后笔者结合对于百年色谱与千年中医药文化之现代化交织的感悟,对色谱技术在此领域的前景做出了展望。

## Abstract (en)

Chromatography is an important branch of analytical chemistry that focuses on the separation and analysis of complex structures. Following more than 100 years of development and improvement, chromatography theory and technology have gradually become sophisticated. It has become a coalition of science, technology, and art. Recently, chromatography has been successfully used in combination with mass spectrometry, nuclear magnetic resonance spectroscopy, and atomic emission spectroscopy. Chromatography and the combination with other techniques has significantly improved the analysis of complex systems, such as the environment, food, petrochemicals, biological specimens, and medicine. As one of the oldest healing systems, Traditional Chinese Medicine (TCM) has served to maintain the health of people in China and worldwide for thousands of years. Therefore, it has become a core representative of traditional Chinese culture. In the past two years, TCM has been widely used to treat COVID-19, especially in patients with mild symptoms. Recently, Chinese government emphasized the inheritance and innovation of TCM and stepped up efforts to promote its modernization. TCM includes herbal medicine, acupuncture, moxibustion, massage, food therapy, and physical exercise, such as Tai Chi. In most cases, the patients are administered a mixture of TCM formulas containing more than two herbal medicines, resulting in a highly complicated compound mixture. There is no doubt that long-term clinical practices have demonstrated the safety and therapeutic effect of TCM. However, the compound mixture must be simplified to identify the active compounds. This is mainly because of the existence of carcinogenic compounds, pesticides, and heavy metal residues introduced through plantation and production processes. Moreover, enzymes within the human system generate further new compounds in response to the entry of the TCM containing thousands of components. Consequently, the complex TCM and organism systems interact with each other, constituting a giant complex drug-organism system. The analysis of this giant complex system is acknowledged as a key aspect in the modernization process of TCM. In the last 20 years, many studies have been conducted to screen and identify effective compounds in TCM. These effective compounds can be either the original compounds or new metabolic components generated in vivo. All these efforts are aimed at simplifying the components of TCM and elucidating the therapeutic mechanism. It is well known that chromatography can provide technical support for complex systems owing to its unique advantage of outstanding separation and analysis capabilities. Therefore, chromatography and its combination with other technologies have become mainstream technologies for promoting the compilation of molecular structure, information, digitalization, and modernization of TCM. This paper reviews the research and application of chromatography and combination technologies in a giant complex TCM formula-organism system. Furthermore, the authors briefly introduce and summarize the understanding, research ideas, and activities of the authors’ team on the modernization of TCM. “Liang Guanxi” and “He strategy” are proposed as novel strategies for studying the giant complex drug-organism system. A distinguished technology integrated with mathematical model of causal relation, combined receptor chromatography, identification of chemical molecular structure and evaluating of pharmacological activities was established. It was successfully employed to determine the core effector-response substances of “Liang Guanxi” herb pairs in a giant complex drug-organism system. Subsequently, utilizing the proposed technology of Combination of Traditional Chinese Medicine Molecular Chemistry, the author’s team designed and developed four series of innovative drugs. Inspired by the hundred years of chromatography history and thousands of years of TCM culture, the future development of chromatographic technology is expected. Furthermore, the mechanisms of TCM in medical healthcare, prevention, and treatment of diseases are likely be explained through chromatography, leading to a new strategy to realize the molecularization and digitalization of TCM, which is beneficial to the development of original new drugs.

纯净是许多人、事物追求的极致,而色谱就是将复杂体系物质进行纯净化而产生的一门集科学、技术与艺术于一体的学科。

色谱作为一门以分离分析为主的学科,历经100余年的发展,由19世纪下半叶科学家发现吸附现象,到1938年薄层色谱的出现,再到气相色谱、液相色谱乃至当今的超高效液相色谱(UHPLC)或超高流速液相色谱(UFLC)及超临界流体色谱、全二维色谱等^[1]^,色谱理论及技术日臻完善。由于传统色谱方法的柱容量和检测器的灵敏度有限,因此结构相近以及低丰度物质的分离、检测和结构解析存在着比较大的困难。色谱联用技术的产生为分析领域带来了跨越性发展。近年来,色谱-质谱联用技术、色谱-核磁共振波谱联用技术、气相色谱-原子发射光谱检测器联用技术、毛细管电泳-质谱联用等新技术的涌现,使色谱在环境、石油化工、食品、生物医药等多个领域得到了广泛的应用^[2,3,4,5]^。

中医药是我国传统文化瑰宝,历经数千年临床实践积淀,疗效经久不衰,在抗击新冠肺炎疫情中,中医药取得了有目共睹的效果。我国2017年颁布了《中华人民共和国中医药法》,制定了一系列方针政策,旨在推动中医药的传承与发展。国务院先后发布了《中共中央国务院关于促进中医药传承创新发展的意见》《中医药发展战略规划纲要(2016-2030年)》及《关于加快中医药特色发展若干政策措施的通知》等系列政策文件,强调“正确把握好继承和创新的关系,充分利用现代科学技术和方法,推动中医药理论与实践不断发展,推进中医药现代化”等。与此同时,由于中药本身是个多成分复杂体系,面临疗效组分与作用机理不明确、部分中成药被屡报安全问题等,严重影响了中医药的国际化,因此中医药的现代化挑战重重。2014年,国家聚焦重大战略需求,整合“973”计划、“863”计划等,启动了国家重点研发计划,着力解决国民经济和社会发展各主要领域的重大科技瓶颈问题,解决制约我国科技计划引领带动创新发展的深层次重大问题,并将“中医药现代化”列为重点研发专项。

色谱理论及技术的迅猛发展,为中医药研究奠定了分离、提取纯化、现代药理、制备工艺和分析的基础^[6]^,对中医药的现代化进程起到了巨大的推动作用。

## 1 色谱技术在中药复杂体系研究中的应用

中药是由数以万计的不同种类的分子组成,其疗效的发挥也与其所含物质息息相关。因此,中药的分子化已成为中药现代化研究的关键制约因素和发展趋势,这恰与色谱技术的优势相吻合,使其成为上述研究领域中的主流手段。

中药的化学成分研究的经典方法即应用柱色谱、薄层色谱等技术对中药复杂提取液进行逐步的分离、纯化、鉴定等。随着分析科学的不断发展,广大色谱分析者在中药复杂成分的分离和检测提升方面开展了大量的创新性研究工作,宋月林等^[7]^建立了非手性-手性色谱-预测多反应监测法,从毛前胡中鉴定出60个化学成分,其中8对香豆素对映异构体得到了良好分离;屠鹏飞等^[8]^建立了在线加压溶剂提取-超高效液相色谱-离子阱-飞行时间质谱体系,对草苁蓉化学成分进行快速分析,鉴定出45个化合物,包括10个苯乙醇苷类、14个环烯醚萜苷类以及21个苯丙醇苷类化合物。梁鑫淼等^[9]^提出了“本草物质组计划”,即全面解析中药物质组成、结构和功能,构建本草物质资源库,阐述中药的多组分多靶点整合调节机制,为创新中药发展提供支撑。在此思路指引下,梁鑫淼等^[10]^开展了系列新型色谱分离材料研究,以此为基础开发了生物碱、天然寡糖、糖脂以及蛋白/多肽药物的色谱分离方法,运用亲水色谱技术、全二维色谱技术等完成了姜黄、红花、黄连、桔梗等中药的化学成分研究。李萍等^[11]^提出了“中药等效成分群”,建立了基于化学成分缺失/捕获-谱效集成表征新方法,该方法利用多种色谱分离技术,从中药和复方提取物中特异性敲除或敲入目标组分或者化合物,进而进行药效对比分析,以期从中药和复方的众多成分中发现能基本代表复方药效的等效成分群。

色谱及其联用技术也推动了中药及其中成药的质量控制提升工作,使其不再局限于显微鉴别、理化鉴别、薄层鉴别等技术。自1985年版《中华人民共和国药典》(简称《中国药典》)开始收录色谱法后^[12]^,高效液相/气相色谱方法已成为药物含量分析的基本分析方法,2000年版《中国药典》首次收录了毛细管电泳法。随着色谱填料的优化及与光谱、质谱技术的联用,新的色谱技术如超高效液相色谱、高效液相-质谱联用、高效液相色谱-圆二色光谱(HPLC-CD)等技术不断涌现,分离分析更高效,应用更广泛。如2015版《中国药典》采用液相色谱-三重四极杆质谱(LC-QQQ-MS/MS)技术,利用阿胶、鹿角胶、龟甲胶的特征肽段鉴定,为市场上胶类动物类药材提供了可靠的质量保证。色谱及其联用技术在中药复杂成分分析研究及质量控制中将发挥更加重要的作用。

## 2 色谱技术在复杂生命过程中的辨识应用

蛋白作为生命活动的执行者,其种类及含量的变化在调控生命过程中发挥着至关重要的作用。利用化合物与靶蛋白的高亲和性,实现中药提取物中活性成分的分离和辨识,具有准确度高、特异性好、速度快等特点,极大地推动了中药药效物质基础研究和活性先导化合物的发现。王序等^[13,14]^以蛋白酶和受体等蛋白为靶标,筛选了近百种中药的有效成分。邹汉法等^[15,16]^进一步提出将蛋白固定在色谱填料表面,利用生物亲和色谱方法研究中药活性成分和质量控制。张丽华等^[17,18,19]^的系列研究,提高了膜蛋白组的鉴定数目和覆盖度,筛选出组织和细胞特异性高表达的受体蛋白,而受体蛋白的选择,对于基于受体亲和色谱方法的中药活性成分筛选方法,具有至关重要的意义。这些研究工作为基于生物亲和色谱的中药活性成分筛选鉴定打下了坚实的理论和技术基础。

许国旺等^[20]^提出了以活细胞为基质的活性成分鉴定方法。在此基础上,又有研究者建立了基于中空纤维细胞捕获^[21,22]^、细胞捕获-分散液相微萃取以及活细胞固相色谱^[23]^等筛选方法。这类方法通常需要破坏细胞的完整性,细胞中内源性物质的释放增加了活性成分鉴定的难度。贺浪冲等^[24]^将高效液相色谱、细胞生物学和受体药理学相结合,在国内率先建立了固定化细胞膜亲和色谱技术。该技术使用液相色谱分离方法,在动态条件下研究活性物质与细胞膜受体的相互作用。至今,固定化细胞膜色谱法已被成功应用于数十种中药和复方的活性成分辨识研究^[25,26,27]^。由于细胞膜表面天然表达了上百种受体蛋白^[28]^,将筛选到的活性成分与某一种受体蛋白建立特异性相互作用网络,存在着一定的挑战。作者研究团队通过将内皮素A受体(ETAR)、*β*_2_肾上腺素受体(*β*_2_-AR)、*α*_1_A-肾上腺素受体(*α*_1_A-AR)、血小板受体(P2Y12)和电压依赖性阴离子通道蛋白(VDAC)等蛋白固定在色谱填料表面,建立了单一和多靶标受体亲和色谱分析方法,用于复杂体系中药物活性成分的高效、特异性和高准确筛选和鉴定^[29,30,31,32,33,34,35]^。常规亲和色谱法常使用大内径色谱柱,靶标蛋白或酶的用量较大,不太适合来源困难、成本高的靶标蛋白,如血管紧张素转化酶和蛋白激酶等。江正瑾等^[36]^及康经武等^[37]^通过引入整体柱固定化酶技术,实现了中药活性成分的高灵敏、低成本筛选鉴定。受体和细胞膜亲和色谱技术不仅可实现活性物质的快速筛选鉴定,还可以进行结合动力学研究,但是靶标物质的固定化在一定程度上影响其空间构象,为了尽可能模拟生理环境,李绍平等^[38,39]^构建了基于自由靶标的生物特异萃取结合高效液相色谱分离方法。该方法将中药和复方提取物与细胞膜或者细胞在缓冲溶液体系中进行孵育,利用超滤、透析等方式去除不结合的化合物,使用高效液相色谱仪有效辨识中药和复方的活性成分。该方法在活性物质的结合动力学以及亲和常数研究方面,尚存在一定的不足。

综上,在基于生物亲和色谱方法的中药活性成分辨识研究中,色谱基质经历了从活细胞到细胞膜,最终到纯化后的受体、蛋白和酶的发展历程。通过靶标物质的不断简化,构建了清晰明了的靶标蛋白-中药-活性成分的网路化和信息化数据库。另外,生物亲和色谱方法鉴定到的是与靶标物质在体外环境下,具有一定亲和作用的化合物,尚需进一步的活性验证及在体研究。

## 3 药物-机体复杂巨系统中色谱技术的应用

中药是一个多种类多成分综合作用的复杂体系,在机体复杂内环境作用下发挥多靶点联合作用,并由此形成了药物-机体、药物-药物、代谢物质群相互作用的药物-机体复杂巨系统,给中药现代化研究带来了重重困难。色谱及其联用技术的优势使其成为上述复杂巨系统研究的主要手段。同时上述复杂巨系统中所产生的数据信息往往是海量的,数学、统计学的思维及技术在此方面起到了至关重要的作用,色谱技术与数理统计学的整合分析极大地推动了药物-机体复杂巨系统中的蕴藏海量信息的数字化与信息化进程(见图1)。

**图1:**
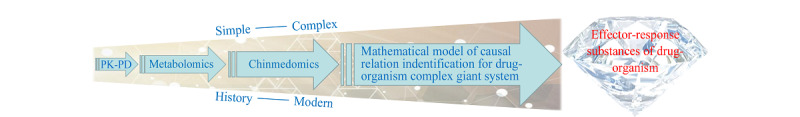
药物-机体复杂巨系统研究模式

1979年,Sheiner等^[40]^建立了一个同时描述药代动力学和药效学的模型并应用于西药的研究,随后该模型被逐渐完善用于中药的药效成分研究,即运用色谱及其联用技术对复方化学物质组的体内过程进行监测,将其与对生物机体的效应关联起来,以寻找中药复方药效物质基础。该方法在药物筛选、药物毒性预测、临床实验设计和剂量方案选择方面做出了突出的贡献。然而以中药复方为代表的药物-机体复杂巨系统中成分种类复杂、数量庞大,同时发挥药效的成分可能是结构不明确的痕量成分或者代谢物成分,这就给上述技术带来了极大的挑战。

20世纪末以来,基因组学、蛋白组学、代谢组学等技术进展迅速,药物-机体复杂巨系统中所蕴含的海量数据被挖掘展现出来,上述组学及其组合多组学技术(泛组学)也被作为主流技术广泛应用于疾病机理及药物作用机制的研究^[41,42,43]^。代谢组学等组学常规数据处理方式侧重于从内源性物质变化差异性的规律中分析病理病机,而忽略了数据之间的相关性尤其是因果相关乃至互为因果相关,使得统计信息中药物成分相关信息有限。

面对上述中药/中药复方-机体复杂巨系统,王喜军^[44]^发展并提出了“中药血清药物化学”研究思路,即运用色谱技术乃至多维色谱技术对血中移行成分及其药动学进行分析,以阐释药物在体内的相互作用,旨在寻找中药复方药效物质。继而王喜军等^[45]^融合代谢组学技术提出了“方证代谢组学”中药-机体研究策略,并运用UPLC-MS技术结合模式识别技术等展开了“四君子汤”“茵陈蒿汤”和“生脉散”等中医经典方的体内药效物质及质量标志物研究,对其体内静态代谢数据进行皮尔逊相关性分析,发现了异秦皮素、京尼平苷等9个化合物可作为茵陈蒿汤治疗湿热黄疸病的质量标志物,五味子素、五味子醇甲、人参皂苷F1等8个化合物可作为生脉散治疗阿尔茨海默症的质量标志物^[46,47]^。该研究强调了物质之间的相关性,能够初步捕捉药物有效成分与内源性应答物质的相关性。然而由于时间因素的忽略,该研究体系中药物-机体协同作用机制及深层次因果关系难以被刻画,因此药物-机体复杂巨系统中核心效应物质辨识仍有待完善和提高。

“万法皆空,因果不空”,因果关系是贯穿于万物发展过程中的永恒规律,尤其对于复杂巨系统的简化、解析至关重要。因果关系分析基本思想最早体现在Wiener^[48]^的构思中:一个时间序列模型的预测效果如果通过加入另外一个时间序列的相关信息而得到改善,说明后者对于前者有“因果影响”。Granger^[49]^进而在线性回归模型背景下将其严格化,认为一般的时间序列会在一定频率范围内发生振动,因此,“因果关系”的“谱表示”尤其重要,为因果关系分析研究奠定了较好的基础,Granger于2003年获得诺贝尔奖^[50]^。在此基础上,Pearl等^[51]^将因果分析、图模型及结构方程进行了综合分析与应用。笔者研究团队基于钱学森先生的开放复杂巨系统、Prigogine的耗散结构理论以及Haken的协同理论,充分考虑中药复方成分、内源性物质、代谢产物体内变化的时间因素,专注于对生物样本体内代谢的动态数据(时间序列)进行分析,将静态相关性变为时间序列之间动态相关性,以因果关系为切入点,捕捉序列间的单向以及互为因果关系,变无方向的相关性为有方向的因果相关,构建了一套因果相关数理模型辨识技术,从而辨识“药物-机体”协同起效的物质基础。运用此模型研究开展了复方丹参方体内代谢研究,并确证了体内的核心效应成分丹参素异丙酯(3-(3,4-二羟基苯基)-2-羟基丙酸异丙酯,isopropyl 3-(3,4-dihydroxyphenyl)-2-hydroxypropanoate, IDHP)^[52]^。

## 4 笔者研究团队对于药物-机体复杂巨系统的思考及创新药物研发

创新药物是解除疾病、保障人类健康的主要途径,中药具有完整的理论体系、确切的临床效果和悠久的应用历史,在疾病治疗及预防方面发挥了不可估量的作用。随着经济全球化、科技进步和现代医学的快速发展,我国中医药也进入快速发展阶段,党中央、国务院把中医药发展上升为国家战略并指出,要围绕国家战略需求及中医药重大科学问题,加快中药新药创制研究。中医药的“创智创新”,具有后续研究和开发应用价值,中医药已成为创新药物研究与开发的重要源泉。

笔者研究团队开展了20余年的中药复方体内效应物质及创新药物研究。面对上述药物-机体复杂巨系统,笔者研究团队先后提出了“君-使”对药、“良关系”药对等新思路^[53]^,驭繁就简地进行中药复杂体系研究;同时发展了化学分子辨识、数理模型辨识、药理功能辨识及临床功效为一体的中药有效成分群辨识技术,以推动中药复方-机体复杂体系的分子化、数字化、信息化;更为重要的是,笔者研究团队基于中医药君臣佐使配伍理论及西医药优势,提出了“组合中药分子化学”^[54]^及“合策略”创新药物分子设计新思路,将中药原成分数据、人体物质数据、数字化、代谢数据信息化,形成君、臣、佐、使分子库集群,运用化学结构-构效辨识、临床方证-功效辨识技术,合成出抗脑缺血化合物丹参素冰片酯(DBZ)^[55]^、新型抗高血压221S系列化合物^[56]^以及抗癫痫93S系列化合物^[57]^,前期研究发现,以上化合物均呈现活性好、机理明晰、易于制备、工艺可控、高效、安全等特点^[58,59,60]^。上述新药候选化合物陆续获得“十一五” “十二五”“十三五”国家新药创制重大专项的资助、入库与候选(见图2)。

**图2:**
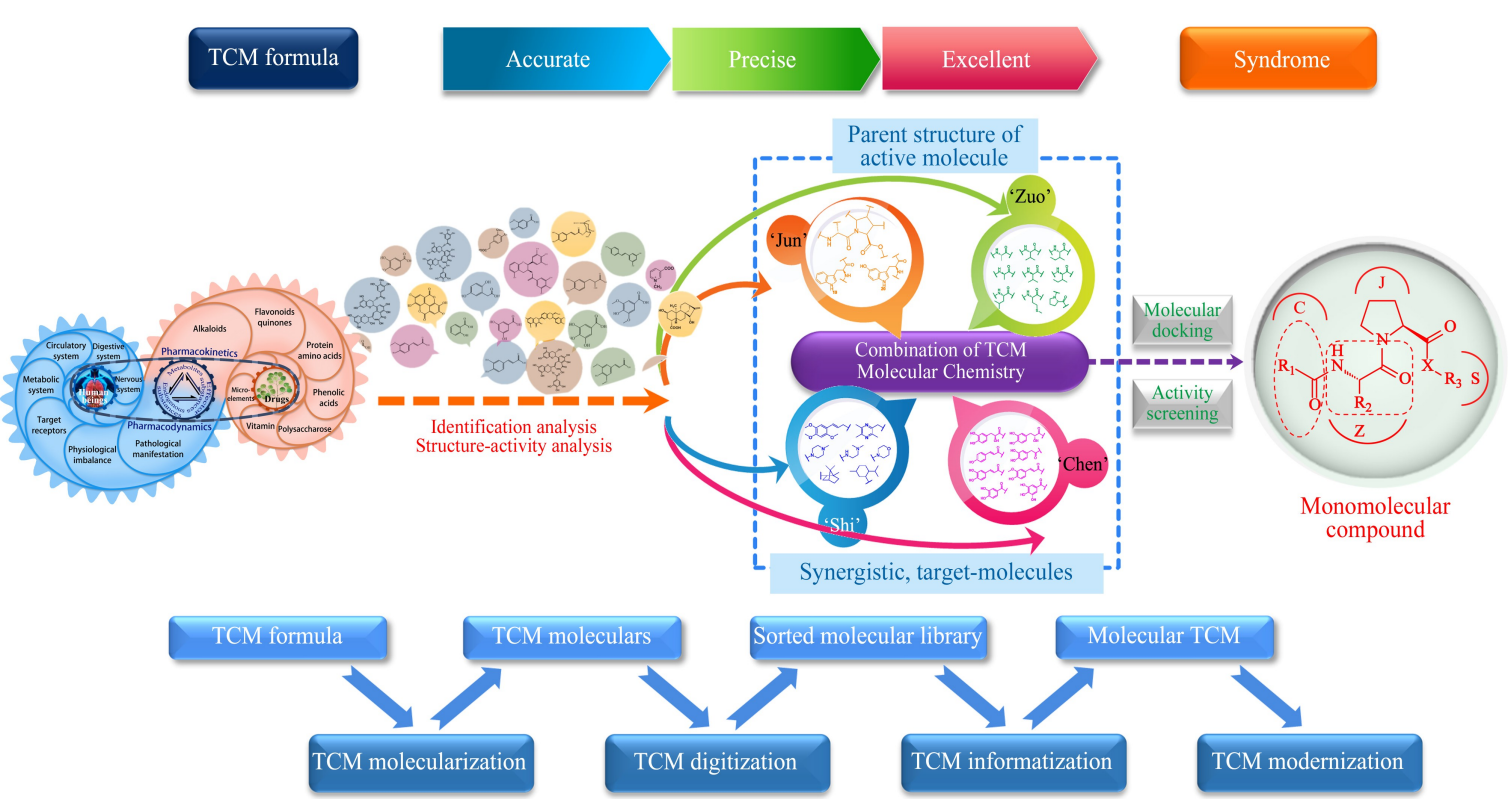
药物-机体复杂巨系统中创新药物的发现和设计思路

以抗心肌缺血1类创新药物丹参素异丙酯为例,复方丹参方^[61]^作为药典名方,由丹参、三七、冰片三味药组成。丹参活血化瘀通经止痛为君药,三七补益气血为臣药,冰片引经报使为佐使药,三药组方,共奏益气活血、标本兼治之功效,用于冠心病、心绞痛等治疗。笔者研究团队运用LC-MS等代谢组学方法对复方丹参方及其中抽提出的“丹参-冰片”“良关系”之君-使对药开展了体内代谢及效应物质研究,从而辨识出复方丹参方及丹参-冰片等药对体内代谢效应物质丹参素异丙酯,并与江苏汉邦科技有限公司联合申报了国家重大科学仪器设备开发专项“超临界流体色谱仪的研制与应用开发”,承担应用超临界流体色谱进行中药组方及其代谢物的研究,完成了丹参素异丙酯及其原料丹参素的手性分离与分析。进而笔者研究团队^[62,63,64]^对该化合物进行了受体色谱功能辨识及抗心脑缺血药理功能辨识,发现其能够保护缺氧复氧的大鼠离体心肌、可明显减轻异丙肾上腺素诱导的心脏纤维化、具有显著改善异丙肾上腺素诱导的心脏重构作用等,可作为心肌缺血等机制引起的高血压、冠心病、心律失常、心衰、瓣膜病、心肌梗死、哮喘及抗高原缺氧等适应症的药物开发。同时,与江南大学蔡宇杰教授合作,利用合成生物学技术与化学合成,开发出单一手性IDHP的制备方法,解决了化学合成法成本高、拆分难度大等问题^[65]^。

## 5 结语

百年色谱与千年中医药的相逢是历史长河的偶然,却也是科学技术与哲学艺术融合的必然,色谱及其联用技术在中医药产业链各环节中均做出了卓越的贡献,对于中医药的“四化”即分子化、数字化、信息化、现代化意义重大,极大地推动了中医药与国际的接轨。中医药理论和中药产业发展有其自身的特殊性,中医药现代化必须同时解决“继承”“发展”与“创新”的难题,才能满足中药现代化、国际化对中药新药创制的基本要求。因此中医药现代化任重道远,分析学人仍需负重前行。

“当下是‘两个一百年’奋斗目标的历史交汇点”,科技创新劲头强劲,色谱工作者正处于当今中国百年未有之大变局的宏大历史背景,应抢抓全球科技发展先机,努力发展色谱领域基础前沿技术,突破传统中医药现代化困境,以继承构筑中医药根本,以创新提升产品质量,以规范促进产业发展,以创智有中国特色的高效创新药物为目标,坚持面向世界科技前沿、面向经济主战场、面向国家重大需求、面向人民生命健康,不断向科学技术广度和深度进军。
